# Books and Pamplets Received

**Published:** 1887-01-01

**Authors:** 


### BOOKS AND PAMPHLETS RECEIVED.

*The Pedigree of Disease;* being six Lectures on Temperament, Idiosyncracy, and Diathesis, delivered in the theatre of the Royal College of Surgeons, in the session of 1881, by Jonathan Hutchinson, F. R. S., late Professor of Surgery and Pathology in the College; President of the Ophthalmological Society, etc. New York: William Wood & Co. 1885.

These Lectures are well worthy of perusal, containing matter of interest on subjects not well understood and especially interesting to the progressive and scientific physician.

*Epilepsy and other Chronic Convulsive Diseases;* their Causes, Symptoms, and Treatment. By W. R. Gowers, M. D., F. R C. P., Assistant Professor of Clinical Medicine in University College; Senior Assistant Physician to University College Hospital; Physician to the National Hospital for the Paralyzed and Epileptic. New York: William Wood & Co. 1885.

The above is an ably written work of 255 octavo pages, in which is considered in detail the etiology, symptoms, etc., of epileptic diseases; the epilepsy after hemeplegia; hysteric epilepsy; hysteroid attacks; certain morbid associations of epilepsy; course of epilepsy; pathology, diagnosis, prognosis, treatment, etc. We note, among the more interesting items referred to in this work, that of borax as an efficient agent in the treatment of epilepsy, also nitro-glycerin, and the mistletoe. The work contains many new and valuable suggestions, and will prove of great practical value to the practitioner.

*A Plea for the Cure of Rupture;* or the Pathology of the Subcutaneous Operation by Injection for the Cure of Hernia. By Joseph H. Warren, A. M., M. D., Member of the British Medical Association; Member of the American Medical Association; Honorary Member of the Vermont State Medical Society; Member of the Massachusetts Medical Society; formerly Surgeon in the U. S. Army, etc. Boston: James R. Osgood & Co. 1884. 104 pp., large, beautiful type, neatly bound and illustrated.

In addition to the author's method of subcutaneous operation, etc, we have in this interesting work a contribution by C. Everett Warren, A. B., M. D., of Boston, on a New Conformateur for measuring, and showing accurately, the contour of Hernia, and deformities of bones, tumors, etc., as well as his paper on Causation of Hernia.

*Two Papers.* By W. H. Daly, M. D., Pittsburgh, Pa., Senior Physician to the Western Pennsylvania Hospital; Senior Physician for Diseases of the Nose, Throat and Chest to the Pittsburgh Free Dispensary.

1. Laryngology and its Cognate Branches in America. Read in the Section of Laryngology, at the eighth International Congress, at Copenhagen, Denmark, August, 1884.

2. The Simplest and Most Efficient Treatment of Diphtheria. Read before the American Laryngological Association, at its eighth annual congress, Philadelphia, 1886.

*Announcement* of Newland's College of Midwifery and Lying-in Institute, incorporated under the Laws of the State of Missouri, December 9, 1884. The only Institution of the kind in the West connected with a Lying-in Institute for the proper and thorough Education of Midwives.

*Information* on Newer Materia Medica, Standard Medical Products, Fine Pharmaceutieal Specialties, Properties and Doses of Drugs. Epitomized for the use of the Busy Physician. Fourth edition, revised and enlarged. From the house of Parke, Davis & Co., Detroit, Mich.

